# Oral Cancer and Oral Potentially Malignant Disorders

**DOI:** 10.1155/2014/853479

**Published:** 2014-05-07

**Authors:** Camile S. Farah, Sook-bin Woo, Rosnah Binti Zain, Alexandra Sklavounou, Michael J. McCullough, Mark Lingen

**Affiliations:** ^1^University of Queensland, Australia; ^2^Harvard University, USA; ^3^University of Malaya, Malaysia; ^4^University of Athens, Greece; ^5^University of Melbourne, Australia; ^6^University of Chicago, USA

## 1. Introduction

Oral potentially malignant disorders (OPMDs) include a variety of lesions and conditions characterized by an increased risk for malignant transformation (MT) to oral squamous cell carcinoma (OSCC) [1]. Leukoplakia and erythroplakia are the most common OPMDs, while special emphasis has been placed on the premalignant nature of oral lichen planus (OLP) [2].

It is generally accepted that the histopathological features of a given lesion, especially the presence and degree of epithelial dysplasia, are currently the most useful indicators of MT risk [3]. However, histopathological assessment alone does not provide an accurate assessment of MT risk, and other features, such as clinical and molecular parameters, must be taken into account. In this regard, the clinical characteristics of OPMDs can show considerable variation within the same histopathologically defined entity that may be critical to the likelihood of progression towards malignancy, thus, serving as prognostic factors of MT and facilitating clinical decisions for further intervention and followup.

Currently, leukoplakia is defined by the World Health Organization as “a white plaque of questionable risk having excluded other known diseases or disorders that carry no risk” [4]. It is a clinical term only and histopathologically may be defined variously from atrophy, hyperplasia, to dysplasia. All frictional disorders (such as chronic cheek biting or benign alveolar ridge keratoses) are excluded by this definition. Two main clinical variants of leukoplakia are recognized: homogeneous leukoplakia with a low risk of MT and nonhomogeneous leukoplakia with a higher risk of MT [5]. The latter can be further subclassified into speckled leukoplakia (red and white but predominantly white), erythroleukoplakia (red and white but probably not predominantly white), nodular leukoplakia, verrucous leukoplakia, and proliferative verrucous leukoplakia [4].

The frequency of epithelial dysplasia, carcinoma-in-situ, or invasive SCC in leukoplakias varies from 8.6% to 60.0% [6–9]. MT of epithelial dysplasia or carcinoma-in-situ occurs in 13.6% to 36.4% of cases [6, 10], and the annual MT rate has been variably reported from 1 to 3% for all leukoplakia [6, 7, 10, 11]. It is well accepted that nonhomogeneous leukoplakia is associated with a higher risk (4- to 7-fold) for MT compared to homogeneous lesions [1–3]. The presence of an erythematous component (erythroleukoplakia) seems to convey a greater risk for MT. This is in agreement with the high malignant potential of pure red lesions (erythroplakia), which, despite its low prevalence ranging between 0.01% and 0.2%, is associated with a very high MT rate which approximates 55–65% in some studies [12]. Furthermore, the frequency of epithelial dysplasia, carcinoma-in-situ, or invasive SCC in erythroplakia is greater than 90% at first biopsy [12]. In addition, proliferative verrucous leukoplakia (PVL), a distinct entity with multiple verruciform white plaques showing a relentless tendency to expand and recur and a predilection to affect nonsmokers and especially women around 50–60 years, has been linked to a MT rate that may eventually approximate 100% [13].

Recently, optical diagnostic aids have been used to better define the clinical features of OPMD and to provide some insight into underlying cellular and molecular changes occurring in these lesions, as highlighted in the paper by Bhatia et al. in this special issue. Light-based devices at various wavelengths have been explored and show promise in assisting the clinician to detect and better visualize OPMD and oral cancer. Surgeons can also use this technology for assessment of tumour margins during surgical resection [14]. During surgical removal of malignancies, surgeons usually remove approximately 10 mm or more of normal appearing mucosal margins with the hope of achieving a margin clearance of 5 mm or more to compensate for fixation shrinkage of the formalin fixed resected specimens. Such clearance has been the routine standard used by surgeons in attempting to prevent recurrence from marginal areas with occult changes. There is thus a dependence on the pathologists' interpretation of surgical close and clear margins which have been used as predictors of tumour recurrence and survival. Despite this, there is still a high recurrence of primary tumours (up to 25%) which may result from the inability to correctly predict the molecular changes already occurring in these margins. This highlights the need to further explore adjunctive methods such as autofluorescence and narrow band imaging in a manner similar to that used for detection of OPMDs. The paper by Diajil et al. in the current issue adds credence to this approach, since laser excision of OPMD as determined by normal operatory light inspection resulted in a significant number of recurrences at the local site, and clinical resolution was most commonly seen with small and intermediate lesions compared to larger sized lesions. Furthermore, as outlined by Kudo et al. in the current issue, histology-based 3D reconstruction of serial tissue sections for evaluating tumour architecture has potential to better inform our understanding of cell invasion at the deep invasive front.

Other than the importance of clinical subtyping of OPMD, the malignant potential of oral leukoplakia and erythroplakia appears to be affected by other parameters, such as site and size. The lateral border of the tongue and the floor of mouth have been correlated with the highest percentage for MT (as high as 44% and 24%, resp.) [1–3]. Despite the limited available data on the prognostic significance of the size of OPMDs, it appears that larger lesions (i.e., greater than 200 mm^2^) are associated with a higher risk (up to 5.4-fold) of MT [1].

Despite the aforementioned MT rates for leukoplakia, it has been known for some time now that so-called “benign hyperkeratosis” transforms to OSCC. As early as 1987, Silverman et al. [6] in the United States noted that 37 out of 235 cases of “benign hyperkeratosis” transformed to invasive carcinoma. Subsequently, Schepman et al. [7] from The Netherlands noted that MT occurred in 6 out of 20 (30%) cases of nondysplastic leukoplakia. Holmstrup et al. [15] in Denmark noted that 2% and 11% of patients with untreated or treated nondysplastic leukoplakia, respectively, developed invasive carcinoma and more recently in 2007 Hsue et al. [10] from Taiwan noted a MT rate of 3.6% in their cases although many of their patients also had submucous fibrosis from the use of betel quid.

Several questions come to mind in this regard. (1) How can benign hyperkeratotic lesions transform to carcinoma? (2) Is there true “benign hyperkeratosis”? (3) Are there any features clinically or molecularly that can help to distinguish between “true” benign reactive keratosis that has no MT potential and nonreactive keratosis that does have a potential for MT? (4) If nondysplastic leukoplakia undergoes MT in at least 4% of patients, does this change the long term management of these patients?

## 2. How Can Benign Hyperkeratotic Lesions Transform to Carcinoma?

There are several factors to consider. Firstly, the diagnosis of dysplasia is notoriously difficult with only, at best, moderate interexaminer agreement between pathologists [16]. As one would expect the discordance is the largest in cases of mild dysplasia and less so in moderate and severe dysplasia. The epithelial changes in mild dysplasia may be subtle or focal and may be attributed to epithelial changes secondary to reaction to injury or inflammation, often termed “reactive epithelial atypia.” This can be observed in biopsies of oral lichen planus, or at the edge of an ulcer. Conversely, cases of reactive epithelial atypia may be overdiagnosed as dysplasia. This is why some pathologists have moved to using a binary system for the diagnosis of dysplasia—low-grade versus high-grade with high-grade dysplasia purportedly more likely to transform to invasive cancer [17]. There is an understanding that low-grade lesions or mild dysplasia may be difficult to distinguish from reactive atypia. Furthermore, architectural abnormalities of dysplasia, such as verrucous configuration without evidence of cytologic dysplasia, are just as important in the evaluation of dysplasia.

Secondly, a single biopsy from a large or nonhomogenous clinical lesion may not be representative. In the study by Lee et al. that evaluated 200 cases, underdiagnosis from a single biopsy versus multiple biopsies was 29.5% and 11.9%, respectively [18]. The prevalence of invasive carcinoma in the resection specimen was 12.0% versus 2.4% in the single versus multiple biopsy cases. This raises another important issue. If within a large or nonhomogenous leukoplakia there are areas that show dysplasia and other regions that show only hyperkeratosis without dysplasia, are the areas of hyperkeratosis without dysplasia precursors to dysplasia?

Thirdly, it may be that leukoplakic areas that show keratosis or hyperkeratosis histopathologically but have little evidence of cytologic abnormality may in fact represent the very earliest changes in carcinogenesis. In two clinically recognised entities, verrucous leukoplakia and proliferative verrucous leukoplakia, the histopathologic changes are those of hyperkeratosis and verrucous epithelial architecture, with only minimal or no evidence of epithelial dysplasia. Proliferative verrucous leukoplakia is a clinicopathologic entity first recognized by Hansen at al. in 1985 [19]. They noted cases of leukoplakia that were “slow-growing, persistent, irreversible, and frequently developed erythematous components.” Studies have subsequently shown that 40–70% of such lesions will develop invasive carcinoma when followed over time [13]. It is very likely that all three factors play a role to a lesser or greater extent, in the transformation of so-called “benign hyperkeratosis” to invasive carcinoma.

## 3. Is There True “Benign Hyperkeratosis”?

Many pathologists use the diagnostic phrase “hyperkeratosis, acanthosis (benign epithelial hyperplasia)” to encompass both frictional keratoses and true leukoplakias without epithelial dysplasia. Lesions of chronic frictional keratosis from parafunctional habits (cheek biting or chewing) and benign alveolar ridge keratoses, common on the retromolar pad, all represent frictional keratoses and will also exhibit hyperkeratosis and acanthosis [20, 21]. As such, when a clinician receives a report of “hyperkeratosis, acanthosis, or epithelial hyperplasia,” without further comment, the lesion could represent an entirely benign lesion caused by friction or a true leukoplakia with the potential of developing dysplasia or invasive cancer. This will confound the results when such lesions are used in leukoplakia research, not as controls, but as lesions of true leukoplakia, possibly early or mild dysplasia. Indeed, many publications have used just such lesions of frictional hyperkeratosis with epithelial hyperplasia under the diagnosis of “leukoplakia.”

## 4. Are There Any Features Clinically or Molecularly That Can Help to Distinguish between “True” Benign Reactive Keratosis That Have No MT Potential and Nonreactive Keratosis That Do Have a Potential for MT?

Clinically, leukoplakias (in particular, homogenous leukoplakias) are for the most part demarcated plaques with a sharp border between the keratotic area and the adjacent normal mucosa, at least for part of the lesion. Although this feature is not present in 100% of cases, it is present in most cases of homogenous leukoplakia and less so in erythroleukoplakia. Homogenous leukoplakia also tends to show shallow fissuring on the surface. At a molecular level, studies have shown a variety of genetic changes, none of which have been consistently noted to be present in dysplasia [22]. It is unclear whether this is due to frictional or reactive keratoses with mild atypia being included in cases of dysplasia.

## 5. If Nondysplastic Leukoplakia Undergoes MT in at Least 4% of Patients, Does This Change the Long Term Management of These Patients?

Presently, there is no consensus regarding the management of leukoplakia, with some favoring a “watch-and-wait” attitude towards dysplasia while others favoring excision [9, 23–25]. Patients with “benign hyperkeratosis” are generally managed with a “watch-and-wait” protocol. One argument made against removal is the high rate of recurrence of leukoplakias. However, if cases of frictional and reactive keratoses are incorrectly included in the group of leukoplakic lesions studied, a high recurrence rate is inevitable. Another argument against removal is that some dysplasia (especially mild dysplasia) regresses. The counter-argument to this is that some cases diagnosed as mild dysplasia may actually represent reactive epithelial atypia from trauma such that regression and recurrence would be expected. Removal of true leukoplakias with a narrow margin with the understanding that a recurrence may occur necessitating a wider excision of the subsequent smaller lesion may be less expensive than repeated biopsies over many years. The followup is also easier since the decision after complete removal is a binary one: absent or present versus is it larger or slightly morphologically altered?

With all these points considered, it is vitally important then to recognize that accurate diagnosis of leukoplakia can only be made when the clinician and pathologist work together and share information.

The malignant potential of OLP, a relatively common, chronic, immunologically-mediated, inflammatory disease has been the subject of significant controversy with conflicting data and opposing views [26–31]. It has been suggested that several reported cases of OLP progressing to OSCC may in fact represent oral erythroleukoplakias with an associated lichenoid inflammatory reaction (so-called “lichenoid dysplasia”). This is particularly noted if such lesions are unilateral and located only at a high risk site such as ventral tongue, but where the histopathology showed a “lichenoid” lymphocytic band [32]. Another explanation is that there is coexistence of two separate processes such as a leukoplakia that develops within typical oral lichen planus. Further, it should be noted that a lymphocytic response to dysplasia and tumor is one of the hallmarks of malignancy [33–35], and can confound the histopathological assessment of these lesions. Nonetheless, most authorities agree today that actual OLP is associated with a relatively low risk for malignant progression.

A large number of studies report that progression to OSCC is more common in the erosive/ulcerative and atrophic OLP, the so-called “red forms”, compared to the “white forms.” An increased risk for MT has also been suggested for the hypertrophic (or plaque-type) form of OLP; the issue with a diagnosis of a pure plaque-form of OLP is how this is different from a leukoplakia. However, the possibility of MT exists in all forms of the disease. Moreover, changes in OLP clinical presentation and severity over time are frequent (e.g., transition from reticular to erosive and/or hypertrophic forms), underlining the need for adequate followup of all cases. It should also be stressed that oral lichenoid lesions, such as those attributed to allergic contact reaction to amalgam or other dental materials and those related to drug-induced allergic reactions, show considerable overlap in their clinical and histopathological features with OLP and may also be linked to an increased likelihood of MT [32].

The frequency of MT in OLP has been reported to range from 0.4 to 5.3% in various studies, usually not exceeding 1% [26–31]. This relatively broad range can be attributed to various factors such as differences in sample size, diagnostic criteria both clinically and histopathologically, follow-up time, assessment of other risk factors, and applied therapeutic interventions. In general, the factors underlining the possible malignant potential of OLP and the significance of related parameters, such as exposure to known carcinogens, remain largely unknown. Recent insights into molecular aberrations in OLP lend further support to the premalignant nature of this condition, which may be related to the effects of chronic inflammatory stimulation. Expression of p-Akt, p-mTOR, and phospho-pS6 has been demonstrated in a subset of OLP cases as outlined by Prodromidis et al. in this special issue, suggesting that activation of Akt/mTOR/pS6 may occur in the context of OLP, possibly contributing to the premalignant potential of individual cases.

Despite the availability of a significant number of cohort studies with an adequate number of patients and an appropriate followup, the exact prognostic significance of the clinical features of OPMDs is not fully appreciated. Large, well-designed multicenter prospective studies with appropriate inclusion and diagnostic criteria and long term followup are in order. Importantly, photographic documentation should be integral to the process. It may be that we need to revisit the classification of OPMD with a view to dividing lesions into “high risk” (leukoplakia and submucous fibrosis) and “moderate-to-low risk” subtypes (OLP, nicotinic stomatitis from reverse smoking, and lupus erythematosus).

OSCC in the oral cavity is a devastating disease and most arise from preexisting leukoplakia. Although clinicians may still struggle to make a decision as to which lesions might become malignant, once these lesions progress to malignancy, the clinical diagnosis of these as oral cancer is, for the most part, reasonably uncomplicated. More than 90% of oral cancers are OSCC and the histopathological diagnosis is also fairly straightforward. The areas of concern would then revolve around identification of microinvasion and superficially invasive SCC. The group of noninvasive verruca-papillary lesions, termed by some as “verrucous hyperplasia” histopathologically and “verrucous leukoplakia” clinically, have also been recognized as a type of OPMD [36]. Controversies exist as to whether some of these verruca-papillary lesions are already malignant in nature, as explored in the paper by Kallarakkal et al. in the current issue.

What is becoming clearer though is the importance of stratifying tumors within the broad family of cancers known as “head and neck malignancies” and “oral cancer.” It is now perfectly clear that this group of tumors is a heterogeneous cluster. It is necessary to pay close attention to this when designing molecular classification profiles. The difficulty in achieving a molecular classification that can be fully utilized for all head and neck cancers is due to the extreme heterogeneity in the genetic expression of these cancers [37]. This marked heterogeneity of their genetic profile is partly due to the fact that head and neck cancers consist of a mixture of cancers of the oral cavity, oropharynx, nasopharynx, hypopharynx, and larynx, known to have varying aetiological factors. Oral cavity cancers are more heterogeneous than other head and neck cancers, and this may be related to different risk habits associated with varied subsites, namely, lip, buccal mucosa and alveolus, and tongue and palatal mucosa. In addition, gene expression microarray studies have found distinct differences in the gene expression profile of oral cancer patients with betel quid chewing habit compared to patients who smoke tobacco [38]. More recently, detailed analysis of HPV positive and negative head and neck squamous cell carcinoma genomes highlights the presence of two distinct groups based on HPV positivity [39] and emphasized the findings of Agrawal et al. [40] and Stransky et al. [41]. High-throughput molecular technologies are currently being used in an effort to further delineate the molecular pathways of cancer and to help with substratification of this large group of heterogeneous tumours that we still term “oral cancer” [42, 43].

As we come to terms with the clinical and histopathological features of OPMD and oral cancer, their molecular features are still largely elusive despite a dramatic increase in the quantity of research being undertaken in this field. Currently the only FDA approved targeted agent for treatment of patients with advanced head and neck cancer is cetuximab (an EGFR-directed monoclonal antibody). Biomarker studies utilising different approaches and samples have been undertaken, and these are highlighted in this special issue by John et al., Prasad & McCullough, and Brooks et al. To advance this research, there is an urgent need for worldwide groups to form research consortia to allow pooling of research materials so as to allow adequate sample sizes to validate current findings and subsequently develop accurate, validated instruments to improve the management and outcomes for oral cancer patients. This type of effort would certainly drive the advancement of therapeutic aspects for head and neck cancers, which currently lags behind cancers from other sites such as breast, colorectal, and lung. With readily available and less expensive molecular diagnostics, a collaborative effort and clarity of terminology and classification, we may unravel the mystery of oral cancer and OPMD and learn more about the sequence of genomic alterations that occur to firstly cause the appearance of a keratosis with minimal or no dysplasia, mild (low-grade) dysplasia, moderate and severe (high-grade) dysplasia, carcinoma-in-situ, and finally invasive carcinoma. This enhanced understanding of the disease process will allow for easier and earlier recognition of MT, better intervention and decreased morbidity and mortality, and ultimately better management for our patients.

*Camile S. Farah* *Camile S. Farah* *Sook-bin Woo* *Sook-bin Woo* *Rosnah Binti Zain* *Rosnah Binti Zain* *Alexandra Sklavounou* *Alexandra Sklavounou* *Michael J. McCullough* *Michael J. McCullough* *Mark Lingen* *Mark Lingen*
